# Sensitivity-driven simulation development: a case study in forced migration

**DOI:** 10.1098/rsta.2020.0077

**Published:** 2021-05-17

**Authors:** D. Suleimenova, H. Arabnejad, W. N. Edeling, D. Groen

**Affiliations:** ^1^ Department of Computer Science, Brunel University London, London, UK; ^2^ Centrum Wiskunde and Informatica, Amsterdam, The Netherlands; ^3^ Centre for Computational Science, University College London, London, UK

**Keywords:** uncertainty quantification, sensitivity analysis, simulation development approach, agent-based modelling, forced migration prediction

## Abstract

This paper presents an approach named sensitivity-driven simulation development (SDSD), where the use of sensitivity analysis (SA) guides the focus of further simulation development and refinement efforts, avoiding direct calibration to validation data. SA identifies assumptions that are particularly pivotal to the validation result, and in response model ruleset refinement resolves those assumptions in greater detail, balancing the sensitivity more evenly across the different assumptions and parameters. We implement and demonstrate our approach to refine agent-based models of forcibly displaced people in neighbouring countries. Over 70.8 million people are forcibly displaced worldwide, of which 26 million are refugees fleeing from armed conflicts, violence, natural disaster or famine. Predicting forced migration movements is important today, as it can help governments and NGOs to effectively assist vulnerable migrants and efficiently allocate humanitarian resources. We use an initial SA iteration to steer the simulation development process and identify several pivotal parameters. We then show that we are able to reduce the relative sensitivity of these parameters in a secondary SA iteration by approximately 54% on average.

This article is part of the theme issue ‘Reliability and reproducibility in computational science: implementing verification, validation and uncertainty quantification *in silico*’.

## 1. Introduction

In recent decades, the use of simulations has become prominent across disciplines, particularly in physics, engineering, economics and computer science. Researchers develop models to investigate, analyse and predict the behaviour and the outcome of real or physical processes and systems [1]. Specifically, they mimic and vary the complexity of different phenomena using input parameters and conceptual models, which include explicit specification, assumption and structure for simulations. However, input parameters and conceptual models are subject to data and model uncertainties which cast doubt on the validity of the simulation output. According to Lin *et al.* [2, p. 4], ‘Uncertainties may have many different sources or drivers. Some of these uncertainties are model related and some are parameter related’. The model uncertainty is due to the assumptions made in the mathematical formulation of the model. In addition, this model can have multiple empirical parameters which have an unspecified or obscure nature. To overcome these uncertainties in simulations, researchers quantify uncertainty by applying uncertainty and sensitivity analysis that are closely related but distinct from each other. In particular, uncertainty analysis (UA) focuses on analysing uncertainty in the output derived from uncertainty in inputs without distinguishing responsible parameters or assumptions in a model. While sensitivity analysis (SA) identifies the relationship between uncertainty in the output and individual inputs of a model to understand which input parameters or assumptions have an impact or influence on the simulation output [1,3,4]. Thus, we need to understand the sensitivity of the predictions to these uncertain parameters, in order to identify which parameters are important for model improvement. We also simplify, refine or eliminate parameters that influence simulation outputs to increase the robustness and reliability of simulations.

In this paper, we focus on SA and investigate how SA of model parameters can help us in developing more detailed simulations, instead of performing traditional model calibration. We propose to guide the development of simulations through iterative SA of key assumptions. We call this approach sensitivity-driven simulation development, or *SDSD*. By modifying the level of detail of simulation against sensitivity, instead of the parameters directly against the error, we argue that we can improve the accuracy of simulations with a reduced risk of over-fitting. We avoid over-fitting by not aiming for a minimal error to begin with, but for a more balanced distribution of parameter sensitivities. Consequently, we are not fitting parameters directly to validation data, and are therefore not affected by over-fitting in that context when we use SDSD.

When modelling a complex system, such as human decision making, we should see a disproportionately large sensitivity of relatively trivial elements of the system, as an indicator that our modelling approach does not accurately balance the importance of the main influencing factors. As such, sensitivity analysis can guide us in simulation development for complex systems. That being said, it is possible for some complex systems to be dominated by a relatively simplistic element (for instance, Newton’s Law of gravity is simple to formulate, but has a dominant effect in star cluster simulations), especially when these systems are guided by first principle physical laws.

Here we apply the proposed SDSD approach to a simulation of forced migration, since accurate predictions of forced migration can help governments and NGO’s in making decisions as to how to help refugees, and efficiently allocate humanitarian resources to overcome unintended consequences [5]. In the current research, model development is guided largely by (a) a top-down design process (e.g. the planned implementation of natural laws in a discretized domain representing an aspect of the physical world) [6], (b) the incremental refinement of existing models (adding in desired aspects that are missing in the original), (c) the availability and incorporation of data sources as inputs or validation targets, or (d) the calibration of existing model parameters against data, in an attempt to further reduce the forecasting error [7].

We present our simulation development approach (SDA) for forced migration, which focuses on approximating human behaviour, and we find ourselves frequently constrained by our limited awareness of natural laws, as well as the lack of existing models that are both relevant and validated. In addition, we have concerns that minimizing the validation error by calibrating existing model parameters against data could lead to over-fitting. Over-fitting not only reduces the ability of our simulations to be re-used in new contexts, but also makes it highly sensitive to the (often incomplete) validation data sources that we work with. As a result, we currently rely mostly on incorporating data sources as model input (approach c), and combine those with heuristics about human behaviour derived from general knowledge and qualitative data.

This paper is organized as follows. In §2, we describe in detail our SDSD approach and present our SA approach to analyse and identify which parameters/assumptions are pivotal in simulations (§3). In §4, we describe forced migration simulation and the Flee agent-based code predicting the distribution of incoming forced migrants’ movements, as well as providing the main model parameters and assumptions within the approach. In §5, we implement the proposed SDSD approach to forced migration case and perform the first iteration of sensitivity analysis on input parameters of Flee. We refine the model assumptions, introduce an updated ruleset for forced migration simulations and perform the second set of sensitivity analysis for comparison purposes (§6). Lastly, we discuss our results and provide conclusions in §7.

## 2. Sensitivity-driven simulation development

**Figure 1.**
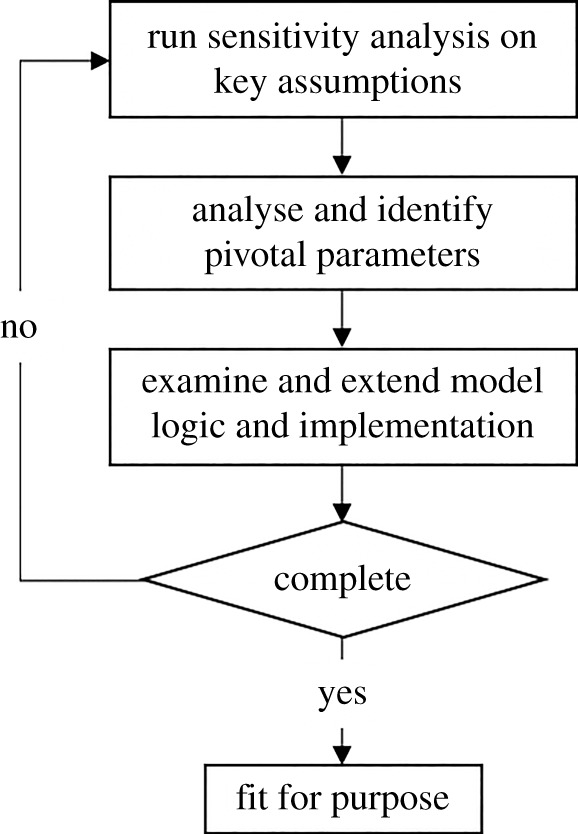
A sensitivity-driven simulation development approach flowchart to perform sensitivity analysis given an existing simulation.

We propose a sensitivity-driven simulation development (SDSD) approach to guide simulation development using sensitivity analysis. Our SDSD approach can be used to further develop and refine existing (prototype) simulations. Given an existing simulation, we can apply it by performing the following steps (figure 1):

(1) We measure the sensitivity of key assumptions in a simulation, using existing sensitivity analysis techniques.
(2) Using the SA results, we identify which parameters have the largest (and possibly even a disproportional) effect on the validation results. We label these parameters as *pivotal parameters*, as small differences in their value have a leveraging effect, leading to much larger, and possibly unrealistic changes to the results.
(3) We examine the underlying model logic that involves the pivotal parameters, and manually extend the model and the implementation such that these parameters are simulated in more detail, and ideally the original parameter is distributed across a larger number of new parameters.
(4) Lastly, we either consider the simulation to be fit for purpose, or go back to the first step and repeat this procedure once more.

Note that step 3 can be done for instance by adding additional rules, making a more detailed breakdown of object types, or incorporating derivative parameters. Listing all the ways that this can be done is beyond the scope of this paper, but we will provide several examples as part of our case study.

It may also be possible to use a variation of this approach to *reduce* the complexity of simulations with minimal impact on the validation error. In this case, one should select the parameters with the lowest sensitivities in step 2, and then proceed in step 3 to see if the rules involving these parameters could be simplified, if several of those parameters could be merged, or if any of them could be replaced with constants. This may then result in a modelling solution with similar validation performance, but with reduced cognitive complexity (and possibly computational cost). We do not showcase this particular variation here, but do consider it a valuable opportunity for future research.

## 3. Sensitivity analysis methods

SA allows us to study how variations in the input parameter or set of parameters contribute to the simulation output. Importantly, SA provides a deeper understanding of the input parameter sensitivity in relation to a given quantity of interest to reduce parameter uncertainties in the most constructive manner. Hence, the SA process aims to understand the relationship between the input parameters and the simulation output, determines parameters contributing to the outcome, identifies the influential input parameters and guides the refinement or future of simulation experiments.

SA may be divided into two broad categories, namely local and global. The first category is local SA, which evaluates information about the simulation behaviour around a selected point in the input parameter while keeping all other parameters constant. The second is global SA taking into consideration and varying the entire range of the input parameters simultaneously. SA had been largely studied and many approaches have been proposed within these categories [8]. In this paper, we primarily focus on global SA using Sobol’s method to determine input parameters (or groups of parameters) mostly responsible both qualitatively and quantitatively for the uncertainty in the simulation output.

### (a) Stochastic collocation and Sobol sensitivity indices

We apply the stochastic collocation (SC) method to perform SA [9], which generates a polynomial approximation $\tilde{q}$ of a quantity of interest *q* in the stochastic space $\xi =(\xi_{1},\ldots ,\xi_{d})\in R^{d}$ via the following expansion:

3.1 $$\begin{array}{rl} & q(\xi )\approx \tilde{q}(\xi )=\sum_{j_{1}=1}^{m_{1}}\cdots \sum_{j_{d}=1}^{m_{d}}q(\xi_{1}^{\left(j_{1}\right)},\ldots ,\xi_{d}^{\left(j_{d}\right)})L_{1}^{\left(j_{1}\right)}⊗\cdots ⊗L_{d}^{\left(j_{d}\right)}(\xi_{1},\ldots .\xi_{d}),\\ & L_{i}^{\left(j\right)}:=\prod_{\begin{array}{c} k=1\\ k\neq j \end{array}}^{m_{i}}\frac{\xi_{i}-\xi_{i}^{\left(k\right)}}{\xi_{i}^{\left(j\right)}-\xi_{i}^{\left(k\right)}}. \end{array}$$

For each input $\xi_{i}\in \xi$, an independent probability density function (pdf) must be provided: *ξ*_*i*_ ∼ *p*(*ξ*_*i*_), *i* = 1, …, *d*, as well as a corresponding univariate set of points $\xi_{i}^{\left(j\right)}$, *j* = 1 …, *m*_*i*_, see [10]. The multivariate interpolant $\tilde{q}$ is constructed using a tensor product of the univariate points, such that $q(\xi_{1}^{\left(j_{1}\right)},\ldots ,\xi_{d}^{\left(j_{d}\right)})$ is the code output evaluated at one point of the *d*-dimensional tensor product. Note from equation (3.1) that these outputs are interpolated to an arbitrary point ξ via a tensor product of one-dimensional Lagrange polynomials $L_{i}^{\left(j\right)}$.

The tensor-product construction leads to an exponential increase in the number of required code evaluations *N*, with increasing dimension *d*. Specifically, *N* = *m*_1_ × · · ·*m*_*d*_, or *N* = *m*^*d*^ if all inputs receive an equal number of points (which is the case in this article). That said, for a moderate number of variables, it is known [11] that the SC method can display exponential convergence, therefore requiring fewer samples than Monte Carlo sampling for the same error.

We use the SC expansion as it is amenable to variance-based global sensitivity analysis and able to obtain estimates of the well-known Sobol sensitivity indices from equation (3.1). Sobol indices are variance-based sensitivity measures of a function *q*(**ξ**) with respect to its inputs $\xi \in R^{d}$ [12]. Just as in the case of the SC method, an independent probability density function *p*(*ξ*_*i*_) is assigned to each parameter *ξ*_*j*_. The Sobol sensitivity method also captures the sensitivity due to higher-order interactions, when several parameters are changed at once.

Let *D*_*u*_ be a so-called partial variance, where the multi-index *u* can be any subset of U:={1,2,…,d}.^1^ Then, the Sobol indices are defined as the normalized partial variances, i.e.

3.2 $$S_{u}:=\frac{D_{u}}{D},$$

where $D:=V\mathrm{ar}[q]=\sum_{u\subseteq U}D_{u}$ [12] is the total variance of *q*. Each *D*_*u*_ measures the fraction of variance in the output *q* that can be attributed to the parameter combination indexed by *u*. By replacing the code output *q* with its SC approximation (3.1), it is possible to obtain an approximation of the *D*_*u*_ as a post-processing procedure, see [9], once all code samples are computed. Importantly, all *D*_*u*_ are positive, and the sum of all possible *S*_*u*_ equals to 1.

The Sobol indices provide values which define the influence of parameters on the output. The closer the value of Sobol indices is to one, the more influential is the corresponding (combination of) input parameter(s). In practice, it is often found that only a subset of parameters yields high first-order Sobol indices (*S*_1_, *S*_2_ etc), and that higher-order interactions tend to be limited. That said, there is no distinct value to differentiate influential from non-influential. However, the relative size of the Sobol indices gives a clear ranking from most to least influential input parameter.

## 4. Forced migration simulation

As an exemplar application in this paper, we apply our SDSD approach to a simulation of forced migration. A comprehensive understanding of human migration provides background knowledge for exploring and understanding the recent increase in forced migration. There are various and often complex reasons behind the decision of people to move, but motivation, desire and pressure are key in most cases [13]. Davenport *et al.* [14] state that forced displacement occurs due to internal or external conditions that trigger the movement of people. In this division, internal conditions include domestic threats of violence, famine and natural disasters, whereas external comprises colonialism, inequality and deterioration of the environment. These factors overlap in forcing people to displace internally and internationally.

However, researchers do use these theories and models to identify the determinants of migration, to explore the migration consequences, to understand the experiences of forced migrants at the destination country and to examine changes in policy decisions concerning population movements [15]. Researchers also attempt to forecast the migration patterns and predict the population counts. It is crucial to predict forced migration, as accurate predictions can help save lives by allowing governments and NGOs to conduct a better-informed allocation of humanitarian resources. Predicting forced population counts can also be critical for policymakers to regulate migration policies and prepare for future challenges with appropriate schemes.

Computational models have the potential to contribute to a better understanding of movement patterns, and to inform, predict and fulfil gaps within forced migration predictions [5,16]. Hence, they have been widely applied to study displacement processes [17] using agent-based models (ABM). ABM is a computational approach that provides an opportunity to model complex systems and is widely applied to various research disciplines. It is suitable for modelling active objects in relation to time, event or behaviour [18]. Its popularity is in part due to the decentralized nature of the approach, which allows a heterogeneous mix of many agents to act and interact autonomously, in turn leading to emergent behaviours in the system at higher levels. ABM consists of agents interacting within an environment. It has been applied to model problems ranging from small-scale behavioural dynamics to large scale migration simulations [19,20].

Raymer & Smith [21] stress that there are four aspects to consider when modelling forced migration, namely the type of migrants, methods structuring available data, modelling approach and measures of uncertainty associated with data. It is also crucial to consider the course of movement of refugees and internally displaced persons (IDPs), including when they decide to leave, where they choose to flee and whether to stay or flee further from the first destination choice [22]. Thus, ABMs could be applied interactively to assist governments, organizations and NGOs in estimating when and where the forced population are likely to arrive [23], and which camps are most likely to become full in the short term.

### (a) The Flee agent-based simulation

**Figure 2.**
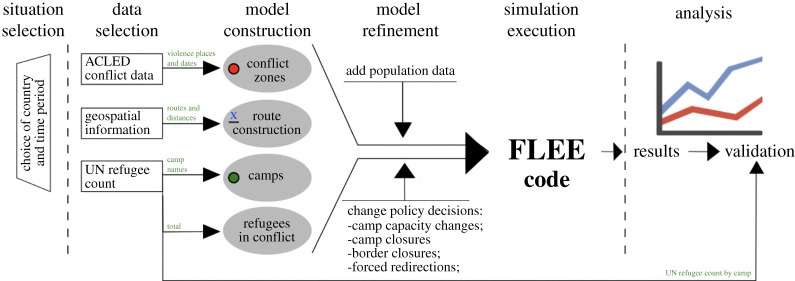
A simulation development approach to predict the distribution of incoming forced population across destination camps [16]. (Online version in colour.)

To simulate the distribution of incoming forced population across destination camps forced to flee because of war, armed conflict and/or political instability, we use a generalized simulation development approach (SDA) which defines the idea of a development process for computational simulation. In the forced migration context, SDA is based on ABM and consists of six main steps, which are situation selection, data collection, model construction, model refinement, simulation execution and analysis (figure 2) [16].

To start with, we select a country and time period of a specific conflict to indicate a simulation time step, which resulted in large scale forced migration. Second, we obtain relevant data to the conflict from three data sources: the United Nations High Commissioner for Refugees (UNHCR, data2.unhcr.org), the Armed Conflict Location and Event Data Project (ACLED, acleddata.com), and mapping platforms, such as the Bing Maps (bing.com/maps) or OpenStreetMap (openstreetmap.org). Third, we construct our initial model using these data sets and create, among other things, a network-based ABM. Once we have built the initial model, we refine it as part of the fourth phase. Here, we manually extract population data to help determine where forcibly displaced people flee from, as well as information on border closures and forced redirection. The fifth phase involves the main simulation, which we run to predict, given a total number of forced population in the conflict and the distribution of displaced people across the individual camps. We run our simulations using the Flee agent-based simulation code (http://www.github.com/djgroen/flee-release) written in Python coding language. Flee is optimized for simplicity and flexibility and provides a range of scripts to handle and convert forced population data from the UNHCR database. Once the simulations have completed, we then analyse and validate the results against the full UNHCR forced migration numbers.

### (b) The Flee parameters and algorithm assumptions

**Figure 3.**
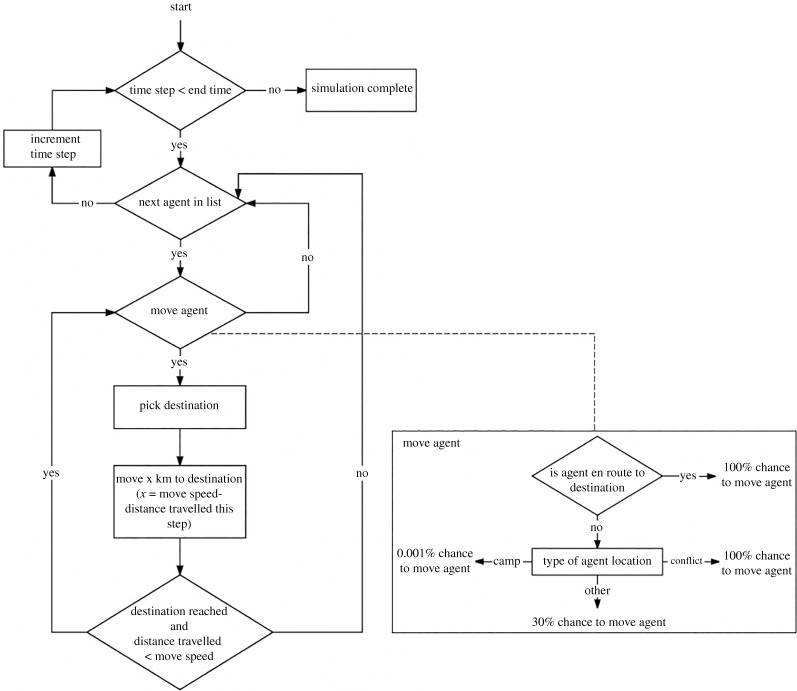
A flowchart of algorithm assumptions in the Flee agent-based code to demonstrate the ruleset predicting forced migrants’ destinations. Move agent component has three location variations expressed by the movement chances [16].

The Flee agent-based simulation code is based on the algorithm assumptions for forced migration including several parameters defining the movement logic of forcibly displaced people (see Suleimenova *et al.* [16] for a more detailed description of the algorithm). Precisely, displaced people are placed in their location of origin, which is one of the conflict locations and they traverse either zero or one link during each time step of a simulation with a speed of up to 200 km per day (i.e. *max_move_speed*). The probability of traversing a link is determined by the movement chance, which is location dependent, such as *camp_move_chance*, *conflict_move_chance* and *default_move_chance*. When an agent traverses a link, it needs to choose one of the available paths. Path selection is made using a weighted probability function, the weight of each link is equal to the attractiveness value of the destination divided by the length of the link in kilometres. A value of 2.0 is the default for camp locations (known as *camp_weight*) making them twice as likely to be chosen as a destination compared to other locations. While a value of 0.25 is the default value for the conflict zone (referred to as *conflict_weight*) making it four times less likely to be chosen as a destination compared to other locations. We illustrate a flowchart of the Flee algorithm ruleset in figure 3 described in Suleimenova *et al.* [16] and present input parameters with descriptions and their default values in table 1, which form logical mechanisms of Flee to simulate the movement of forced migration. We investigate the sensitivity of these parameters as the assumptions are weak and it is important to understand which parameters are pivotal and influential in the simulation output.

**Table 1.** The list of input parameters defining forced migration simulation algorithm.

input parameters	description	default value
*max_move_speed*	agents’ maximum movement speed in the simulation while traversing between locations.	200 km/day
*camp_move_chance*	probability of an agent moving from camp location where an agent resides to another location.	0.001
*conflict_move_chance*	probability of an agent moving from conflict location where an agent resides to another location.	1.0
*default_move_chance*	probability of an agent moving from other (default) location where an agent resides to another location.	0.3
*camp_weight*	the attractiveness value for camp locations making them twice as likely to be chosen as destination.	2.0
*conflict_weight*	the attractiveness value for conflict locations making them four times less likely to be chosen as destination.	0.25

## 5. Sensitivity analysis of the initial Flee ruleset

**Figure 4.**
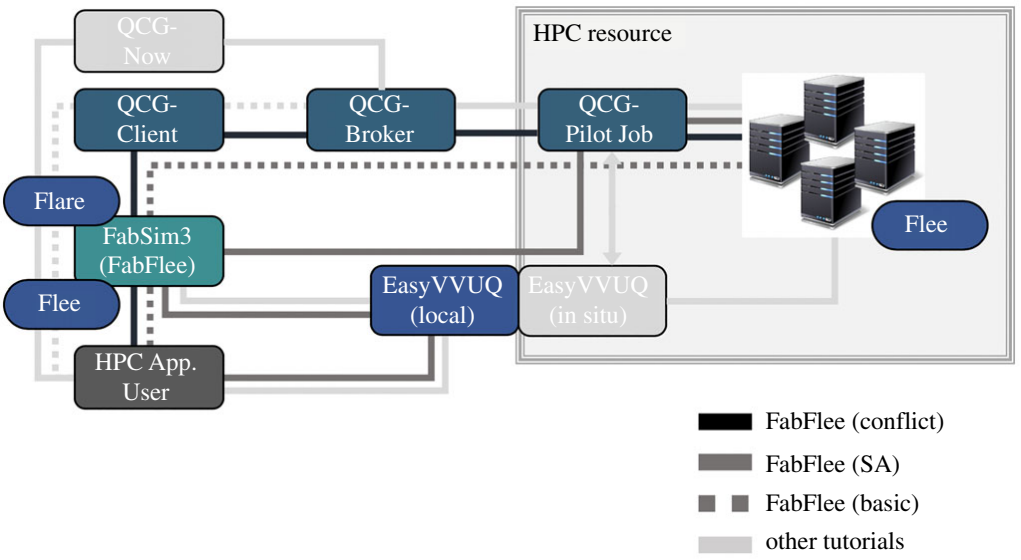
Overview of the FabFlee plugin path in congestion with other VECMAtk components. (Online version in colour.)

For forced migration sensitivity analysis, we use an automated execution environment that constructs highly transparent and customized simulations and achieves automation of key tasks by simplifying and accelerating activities, including construction, execution and analysis of simulations. Specifically, we combine the Fabric for Flee Simulation (FabFlee) plugin (https://github.com/djgroen/FabFlee) [24,25], the EasyVVUQ toolkit (github.com/UCL-CCS/EasyVVUQ) and QCG-PilotJob (https://qcg-pilotjob.readthedocs.io. FabFlee is an integration of Fabric for Simulation (FabSim3, https://github.com/djgroen/FabSim3) toolkit [26] and the Flee agent-based simulation code. Moreover, EasyVVUQ aims to facilitate verification, validation and uncertainty quantification (VVUQ) for a range of simulation, and supports a variety of VVUQ algorithms [27]. To demonstrate, the Sobol’s indices using the stochastic collocation is implemented in EasyVVUQ, and is used for forced migration sensitivity analysis. Furthermore, QCG-PilotJob is a pilot job mechanism that bypasses constraints of regular queuing systems identified with the scheduling of workloads, such as limits in the number of concurrent jobs. These tools are the VECMA toolkit components (http://www.vecma-toolkit.eu) enabling users to include their added values while retaining a limited deployment footprint (figure 4).

### (a) First iteration set-up

To identify the influential (or pivotal) parameters in the Flee algorithm, we perform sensitivity analysis on parameters presented in table 1. First, we define parameter space including the minimum, maximum and default values in the EasyVVUQ script. Second, we specify the range for each parameter and vary using the uniform distribution for sensitivity analysis as shown in table 2 and Chaospy library within EasyVVUQ with a polynomial order of 3 to create a 4 point quadrature rule for each input parameter. Next, we execute EasyVVUQ script on Eagle supercomputer and obtain simulation results for four African conflict scenarios with different simulation periods. We simulate Mali for 300 days from the 29th February 2012, the Burundi conflict for 396 days from the 1st May 2015, the South Sudan instance for 604 days from the 15th December 2013 and the Central African Republic for 820 days from the 1st December 2013. We chose these simulation periods based on conflict progressions that forced people to flee from their origins to neighbouring countries.

**Table 2.** Defining parameter space for the uncertain parameters of forced migration simulation.

parameters	type	min value	max value	default value	uniform range
*max_move_speed*	float	0.0	40 000	200 km/day	(20, 500)
*camp_move_chance*	float	0.0	1.0	0.001	(0.0, 0.1)
*conflict_move_chance*	float	0.0	1.0	1.0	(0.1, 1.0)
*default_move_chance*	float	0.0	1.0	0.3	(0.1, 1.0)
*camp_weight*	float	1.0	10.0	2.0	(1.0, 10.0)
*conflict_weight*	float	0.1	1.0	0.25	(0.1, 1.0)

### (b) First iteration results

We obtain the distribution of forced migrants arriving at neighbouring camps of conflict scenarios at each day for specified simulation periods. The simulation outputs are validated against the UNHCR data. Moreover, EasyVVUQ performs SA and provides the Sobol indices results for six input parameters and we calculate the average difference for each parameter presented in table 3. We observe that the most influential parameters are *default_move_chance* and *camp_move_chance* and are highly sensitive for all four conflict simulations. The *max_move_speed* parameter is also pivotal as the Sobol indices for Mali, South Sudan and CAR are 0.2249, 0.1788 and 0.1617 respectively and greater than 0.05 contributing to the simulation output. *conflict_move_chance* and *camp_weight* have a slight impact compared to earlier mentioned parameters with the average differences of 0.0829 and 0.0628. The only input parameter with the lowest values is *conflict_weight* which is a non-influential variable of for all simulation instances. We also present the Sobol indices for the combination of parameters in appendix B (table 9). We observe that all variations of parameter combinations have the Sobol indices below 0.01 and thus, they do not strongly correlate with each other.

**Table 3.** FabFlee and EasyVVUQ input parameter exploration results for six parameters of forced migration.

input parameters	Mali	Burundi	South Sudan	CAR	average difference
*max_move_speed*	0.2249	0.0485	0.1788	0.1617	0.1534
*camp_move_chance*	0.3427	0.4404	0.0766	0.1945	0.2635
*conflict_move_chance*	0.0739	0.0233	0.0477	0.1869	0.0829
*default_move_chance*	0.2273	0.4121	0.4891	0.2503	0.3447
*camp_weight*	0.0396	0.0526	0.0835	0.0758	0.0628
*conflict_weight*	0.0288	0.0022	0.005	0.0315	0.0168

According to the obtained sensitivity results, we identified three pivotal parameters in the Flee algorithm, which are *default_move_chance*, *camp_move_chance* and *max_move_speed*. We refine these pivotal parameters because in the next section, we describe how we refine identified pivotal parameters in the Flee algorithm to improve the reliability and validity of our simulations.

## 6. Sensitivity analysis of the refined Flee ruleset (Ruleset 2.0)

In this section, we propose and discuss the refinement of assumptions for Flee parameters to provide a higher amount of detail to our simulations.

**Modification 1:** The most influential parameter is *default_move_chance* that we modify by changing agents’ travelled distance. It is reasonable to assume that if an agent has travelled a sizeable distance, it would benefit from a break. However, if the agent has travelled relatively little it is unreasonable to assume that it would remain in a particular non-camp location as it is likely to have relatively good supplies, and is likely unwilling to waste excessive time finding safety. To incorporate the above discussed assumptions, we define a notion of recent distance travelled, called the Recent Travel Distance Index (RTDI), and calculate this using the following ratio:

6.1 $${\text{RTDI}}_{t}=\frac{{\text{RTDI}}_{t-1}+(({\text{dist}}_{t})/(\text{max}\_\text{move}\_\text{speed}))}{2}.$$

Here, RTDI_*t*−1_ is set to 0 when *t* = 0. The threshold value for the RTDI can be set between 0.0 and 1.0, and we chose a threshold value of 0.5. Using this value, when agents set out, they will at least travel for a day at maximum speed before requiring a break. If they travel less than that on the first day, then the RTDI will still be less than 0.5 and the movement chance of the location for agents at that moment will remain 1.0. In this way, we amend the initial rule set without changing the actual parameter of *default_move_chance*. For clarification purposes, a higher RTDI threshold will cause agents to travel longer without breaks, while a lower one will introduce more frequent breaks.

**Modification 2:** We refine the (*max_move_speed*) parameter by proposing a new range value and adding a new mode of transport (*max_walk_speed*) based on qualitative research we have conducted with NGOs and researchers in the field. Specifically, the obtained qualitative data suggest that people walk on foot with a movement speed of 3–4 km per day when they initially depart. It is due to roads being blocked by armed forces and migrants not having a secured good shared transportation at the start of their journey. Moreover, there is evidence that people use shared vehicles in other cases. These vehicles may not arrive immediately, make detours, start and stop to transit other people, and vehicles and roads may not be in optimal condition. Average move speed is 30–40 km per day when agents travel using share vehicles and they travel on average for 12 hours per day.

Based on these findings, we improve the assumptions behind agents’ movement speed by refining the range for *max_move_speed* from 200 km per day to 420 km per day in all travelling cases with vehicles. In the refined iteration, the *max_move_speed* parameter excludes people that travel on foot (that is now covered by *max_walk_speed*). As a result, we changed the minimum value for *max_move_speed* from 20 km per day to 100 km per day (see the uniform range column in table 4). Moreover, the new additional *max_walk_speed* parameter has a movement speed of 35 km per day during the first travel from a conflict zone. Nonetheless, the average speed is affected by both parameters in the refined iteration, and the movement chances of various location types. There is no mechanism in place to keep the average move speed fixed in any of the simulations, because we do not have validation data about this aspect.

**Table 4.** Defining a refined parameter space for the uncertain parameters of the Flee simulation.

parameters	type	min value	max value	default value	uniform range
*max_move_speed*	float	0.0	40 000	420 km/day	(100, 500)
*max_walk_speed*	float	0.0	40 000	35 km/day	(10, 100)
*camp_move_chance*	float	0.0	1.0	0.001	(0.0, 0.1)
*conflict_move_chance*	float	0.0	1.0	1.0	(0.1, 1.0)
*default_move_chance*	float	0.0	1.0	0.3	(0.1, 1.0)
*camp_weight*	float	1.0	10.0	2.0	(1.0, 10.0)
*conflict_weight*	float	0.1	1.0	0.25	(0.1, 1.0)

The *camp_move_chance* parameter is also a pivotal parameter, which we do not refine as it is relatively hard due to little data availability on camp conditions and the duration of migrants’ stay at these locations. We define the range for *camp_move_chance* (0.0–0.1) despite data limitation as there are indications of the camp population remaining relatively stable later in the conflict and people leaving camp locations at a subsequent stage. Thus, the very low end of the range is 0.0 and the upper section of the range is 0.1, which is possible but rather unlikely to be the case in realistic circumstances. Bearing this mind, we have picked an appropriate range for *camp_move_chance* that is not refined in the second iteration.

### (a) Second iteration set-up

In the second iteration of our sensitivity analysis, we change the default value of *max_move_speed* to 420 km/day in the EasyVVUQ script. We include a new parameter *max_walk_speed*, which has an agent’s speed movement of 35 km/day as a default value. The uniform distribution range is also modified for the *max_move_speed* parameter, which starts from 100 km/day and specify the range for *max_walk_speed* varying from 10–100 km/day. The remaining four parameters are unchanged but we improved the algorithm ruleset of Flee as discussed at the start of this section. In table 4, we provide an updated version of parameter space and an iterated version of input distributions for parameters by extending the previous definitions (table 2). We demonstrate the EasyVVUQ script listings for sensitivity analysis in appendix A.

### (b) Second iteration results

We obtain the second iteration results for the distribution of forced migrants arriving at neighbouring camps each day for four African countries with specified simulation periods. EasyVVUQ performs the second iteration of SA and provides new Sobol indices results for seven input parameters and we calculate the average difference for each parameter presented in table 5. In this set of results, we find that the Sobol indices values for the new parameter, namely *max_walk_speed*, is 0.0 for all four countries and, thus, are not influential in the simulation output. However, the Sobol indices for *max_move_speed* are lower than the indices in the previous results, but still have an impact on output. The movement chance of locations vary differently across conflict scenarios and are highly sensitive compared to other parameters. For instance, the Burundi conflict has the highest value for the *camp_move_chance* parameter (0.7242) and the lowest values for *conflict_move_chance* and *default_move_chance* (0.0482 and 0.0447 respectively) against the other three conflicts. On the contrary, the simulation output for Mali is sensitive to all of the move_chance parameters. These variations in the results due to the differences in the simulation periods, network map constructions linking conflict locations with camps and how agents traverse across conflicts.

**Table 5.** FabFlee and EasyVVUQ input parameter exploration results for seven parameters of forced migration using the updated algorithm ruleset.

input parameters	Mali	Burundi	South Sudan	CAR	average difference
*max_move_speed*	0.1367	0.0556	0.1326	0.0837	0.1021
*max_walk_speed*	0.0000	0.0000	0.0000	0.0000	0.0000
*camp_move_chance*	0.3356	0.7242	0.0261	0.1524	0.3095
*conflict_move_chance*	0.2133	0.0482	0.1968	0.4591	0.2293
*default_move_chance*	0.0929	0.0447	0.1619	0.0476	0.0868
*camp_weight*	0.0667	0.0829	0.1558	0.0811	0.0966
*conflict_weight*	0.0835	0.0071	0.0066	0.0444	0.0354

It is also important to note that the average difference for *default_move_chance* in the second iteration (0.0868) is much lower compared to the initial results (0.3447). Contrarily, the *camp_move_chance* and *conflict_move_chance* parameters have higher sensitivity to highlight their importance as we have not refined these assumptions. Similarly, the Sobol indices values for *camp_weight* and *conflict_weight* have slightly increased for all four conflict simulations. Nonetheless, the *conflict_weight* parameter was the only parameter with the lowest sensitivity in table 3, which still holds for Burundi, South Sudan and CAR, but has changed for the Mali conflict. The combination of parameters in the second iteration of sensitivity analysis is still non-influential to the simulation output and we present these results in appendix B (table 10). We observe that all variations of parameter combinations have the Sobol indices below 0.01 and thus, they do not strongly correlate with each other.

## 7. Discussion and conclusions

We have presented an approach to guide simulation development by iteratively performing a sensitivity analysis on the model assumptions, and then refining the underlying algorithm based on the most sensitive parameters (the so-called pivotal parameters). We demonstrated our approach on an agent-based simulation of forced migration, and find that we are able to reduce the relative sensitivity of two parameters (*default_move_chance* and *max_move_speed*) by respectively 75% and 33% (see tables 6 and 7 for the absolute and relative change in input parameters between two sensitivity iterations). We attain these reductions by adding a range of new assumptions and logical mechanisms to our simulation algorithm, and including a single additional parameter (the maximum walking speed). Because Sobol sensitivity indices normally add up to 1.0, the sensitivity of other parameters (excluding *default_move_chance*, *max_move_speed* and *camp_move_chance*) in our model relatively increased as a result. It is due to the choice of decision-making logic in the algorithm affecting the sensitivity of parameters rather than the new additional parameter (*max_walk_speed*), which is not a sensitive parameter and illustrated Sobol scores of 0.0000 for all conflict simulations.

**Table 6.** Absolute change in input parameters between an initial and redefined Flee algorithm of forced migration simulation.

input parameters	Mali	Burundi	South Sudan	CAR	average difference
*max_move_speed*	−0.0882	0.0071	−0.0462	−0.078	−0.0513
*camp_move_chance*	−0.0071	0.2838	−0.0505	−0.0421	0.046
*conflict_move_chance*	0.1394	0.0249	0.1491	0.2722	0.1464
*default_move_chance*	−0.1344	−0.3674	−0.3272	−0.2027	−0.2579
*camp_weight*	0.0271	0.0303	0.0723	0.0053	0.0338
*conflict_weight*	0.0547	0.0049	0.0016	0.0129	0.0185

**Table 7.** Relative change in input parameters between an initial and redefined Flee algorithm of forced migration simulation.

input parameters	Mali (%)	Burundi (%)	South Sudan (%)	CAR (%)	average difference (%)
*max_move_speed*	-39.2	14.6	-25.8	-48.2	-33.4
*camp_move_chance*	-2.1	64.4	-65.9	-21.6	17.5
*conflict_move_chance*	188.6	106.9	312.6	145.6	176.5
*default_move_chance*	-59.1	-89.2	-66.9	-81	-74.8
*camp_weight*	68.4	57.6	86.6	7	53.7
*conflict_weight*	189.9	222.7	32	41	109.8

It is also important to note that our input parameters do not strongly correlate with each other and can be treated as independent random variables as the Sobol indices values are below 0.01 (appendix B). However, there might be instances when the Sobol analysis is less effective for parameters which strongly correlate with each other. To overcome this limitation, we suggest to combine the co-dependent parameters and vary them uniformly in different runs.

We also calculate the mean total error^2^ for two sensitivity iterations and another simulation sets with the refined parameters retaining their default values (table 4) across four African countries. We present the obtained values in table 8 where the mean total errors for sensitivity iterations have decreased after the changes in the logical structure of the algorithm and parameter refinements. For the simulation sets with default values, the total errors are considerably higher for Burundi, South Sudan and CAR, but lower for Mali in comparison with two sensitivity iterations.

**Table 8.** The mean total error values for four African conflict situations comprising two sensitivity iterations and simulations with parameters retaining default values of Flee algorithm.

simulations	Mali	Burundi	South Sudan	CAR
iteration 1	0.4914	0.3832	0.4926	0.3809
iteration 2	0.4101	0.3361	0.4753	0.3257
default values	0.3122	0.2571	0.5234	0.3378

We believe sensitivity-driven simulation development (SDSD) is a new approach that allows us to systematically guide the process of simulation refinement when it is not desirable to directly calibrate a model to existing data (e.g. in cases where generality is paramount, or where the existing data is known to have biases and omissions). It provides us with the advantage of identifying the importance of our assumptions and when done in multiple iterations, may help us to prevent developing models that end up giving radically different results because of minor changes in a single parameter.

However, SDSD itself is of course not without limitations. First, the use of SA in an iterative fashion can be computationally expensive, especially when the number of underlying assumptions, and their dimensionality, is large. Second, the net benefit of adopting SDSD is heavily dependent not only on the SA, but also on the subsequent simulation refinement steps that developers choose to take. These refinement steps cannot be automated, and scenarios are possible where one could be simply unable to extend a single parameter assumption into a more refined and realistic approximation. For instance, a lack of understanding of the underlying simulated problem (also known as *simuland* in some communities) may limit one’s ability to perform such refinements. Third, although we argued that SDSD could be used to guide model *simplifications*, we have not demonstrated this in practice yet, and its benefit for that specific purpose has not been proven at this stage.

In our case, we were able to bypass the first limitation by using supercomputers, and due to the relatively limited complexity of the Flee code. Although not investigated in this paper, one could gain performance benefits by using alternative approaches such as Polynomial Chaos Expansion (PCE), which is a probabilistic approach projecting the simulation output based on orthogonal stochastic polynomials in the random input parameters, or a Quasi Monte Carlo approach, which uses probability distributions of varying parameters and generate output samples to calculate the mean and the variance of QoI. However, the efficacy of either approach should first be compared to a baseline investigation, which we have provided in this paper. We were able to bypass the second aforementioned limitation by liaising directly with organizations that have expertise on the ground, such as the International Organization for Migration. As a result, we were able to demonstrate this approach successfully in this work.

In a world where over-fitting presents a real danger not only to our academic profession, but also to the global community as a whole [28], we argue that SDSD could provide a much less risky route to selectively refining and improving our models. In which areas this will indeed be the case is a matter we need to investigate next.

## Footnotes

1 Specifically, u∈F, where F is the power set of U.

2 For more details on the mean total error calculation see Suleimenova *et al.* [16].

## Data accessibility

All output data will be publicly available on Figshare under a CC-By 4.0 license (after review). (see doi:10.17633/rd.brunel.13525751)

## Appendix A. Defining parameter space and uniform distributions for the EasyVVUQ script

We define the parameter space for the uncertain parameters of forced migration simulation in the EasyVVUQ script as follows:

We vary a set of parameters with corresponding distributions using the stochastic collocation sampler with a polynomial order of 3 for sensitivity analysis of forced migration simulation as shown below:

## Appendix B. Sensitivity results for the combination of parameters

We present the Sobol indices for input parameters and the combination of parameters for both iterations obtained using stochastic collocation method in tables 9 and 10.

**Table 9.** FabFlee and EasyVVUQ input parameter exploration results for six parameters of forced migration.

		Mali	Burundi	South Sudan	CAR
input parameters	max_move_speed (0)	0.2249	0.0485	0.1788	0.1617
	camp_move_chance (1)	0.3427	0.4404	0.0766	0.194
	conflict_move_chance (2)	0.0739	0.0233	0.0477	0.1869
	default_move_chance (3)	0.2273	0.4121	0.4891	0.2503
	camp_weight (4)	0.0396	0.0526	0.0835	0.0758
	conflict_weight (5)	0.0288	0.0022	0.005	0.0315
combination of parameters	1, 2, 3	0.00002	0.00007	0.0008	0.0001
	4, 5	0.00534	0.00001	0.0044	0.0075
	0, 1, 2, 3	0.00004	0.000007	0.00009	0.00003
	0, 4, 5	0.00002	0.000001	0.00001	0.00003
	1, 2, 3, 4, 5	0.00001	0.000007	0.00007	0.00003
	0, 1, 2, 3, 4, 5	0.00002	0.000002	0.00005	0.00002

**Table 10.** FabFlee and EasyVVUQ input parameter exploration results for seven parameters of forced migration using the updated algorithm ruleset.

		Mali	Burundi	South Sudan	CAR
input parameters	max_move_speed (0)	0.1367	0.0556	0.1326	0.0837
	max_walk_speed (1)	0.0000	0.0000	0.0000	0.0000
	camp_move_chance (2)	0.3356	0.7242	0.0261	0.1524
	conflict_move_chance (3)	0.2133	0.0482	0.1968	0.4591
	default_move_chance (4)	0.0929	0.0447	0.1619	0.0476
	camp_weight (5)	0.0667	0.0829	0.1558	0.0811
	conflict_weight (6)	0.0835	0.0071	0.0066	0.0444
combination of parameters	0, 1	0.0000	0.0000	0.0000	0.0000
	2, 3, 4	0.00006	0.000004	0.0007	0.0001
	5, 6	0.0079	0.0002	0.0226	0.0147
	0, 2, 3, 4	0.00006	0.000002	0.0002	0.00001
	0, 5, 6	0.00001	0.000001	0.0001	0.00001
	1, 2, 3, 4	0.000001	0.0000001	0.0000008	0.0000006
	1, 5, 6	0.0000004	0.0000	0.0000003	0.0000002
	0, 1, 2, 3, 4	0.000002	0.0000003	0.000002	0.000001
	0, 1, 5, 6	0.0000008	0.0000001	0.0000007	0.0000005
	2, 3, 4, 5, 6	0.00001	0.000002	0.0002	0.00002
	0, 1, 2, 3, 4, 5, 6	0.00001	0.000002	0.00001	0.00001
